# Incidence and Resolution of Eribulin-Induced Peripheral Neuropathy (IRENE) in Locally Advanced or Metastatic Breast Cancer: Prospective Cohort Study

**DOI:** 10.1093/oncolo/oyad191

**Published:** 2023-08-09

**Authors:** Hans-Joachim Lück, Marcus Schmidt, Tobias Hesse, Oliver Hoffmann, Bernhard J Heinrich, Tjoung-Won Park-Simon, Eva-Maria Grischke, Rudolf Weide, Harald Müller-Huesmann, Kerstin Lüdtke-Heckenkamp, Dorothea Fischer, Cosima Zemlin, Matthias Kögel, Jane Wu, Helga Schmitz, Christian Engelbrecht, Christian Jackisch

**Affiliations:** Gynecologic Oncology, Gynäkologisch-Onkologische Praxis am Pelikanplatz, Hannover, Germany; Department of Obstetrics and Gynecology, University Medical Center Mainz, Mainz, Germany; Department of Gynecology, Agaplesion Diakonieklinikum Rotenburg gGmbH, Rotenburg (Wuemme), Germany; Department of Gynecology and Obstetrics, Universitätsklinikum Essen, Essen, Germany; Hematology/Oncology, Praxis Heinrich/Bangerter, Augsburg, Germany; Department of Obstetrics and Gynecology, MHH Hannover, Hannover, Germany; Oncology, Universitäts-Frauenklinik Tübingen, Tübingen, Germany; Oncological Outpatient Department, Praxis für Hämatologie und Onkologie Koblenz, Koblenz, Germany; Department of Hematology/Oncology, Brüderkrankenhaus St. Josef Paderborn, Paderborn, Germany; Department of Hematology/Oncology, Niels Stensen Clinics, Franziskus Hospital, Georgsmarienhütte, Germany; Department of Gynaecology and Obstetrics, Hospital Ernst von Bergmann, Potsdam, Germany; Department for Gynecology, Obstetrics and Reproductive Medicine, Saarland University Medical Center, Homburg, Germany; Klinikum Worms Frauenklinik, Worms, Germany; Biostatistics, Eisai Inc., Nutley, NJ, USA; Medical Department, Eisai GmbH, Frankfurt, Germany; Medical Affairs, Eisai GmbH, Frankfurt, Germany; Department of Obstetrics and Gynecology, Sana Klinikum Offenbach GmbH, Offenbach, Germany

**Keywords:** breast neoplasms, peripheral nervous system diseases, eribulin, cohort studies, prospective studies, medical oncology

## Abstract

**Background:**

Eribulin, a halichondrin-class microtubule dynamics inhibitor, is a preferred treatment option for patients with advanced breast cancer who have been pretreated with an anthracycline and a taxane. Peripheral neuropathy (PN) is a common side effect of chemotherapies for breast cancer and other tumors. The Incidence and Resolution of Eribulin-Induced Peripheral Neuropathy (IRENE) noninterventional postauthorization safety study assessed the incidence and severity of PN in patients with breast cancer treated with eribulin.

**Patients and Methods:**

IRENE is an ongoing observational, single-arm, prospective, multicenter, cohort study. Adult patients (≥18 years of age) with locally advanced or metastatic breast cancer and disease progression after 1-2 prior chemotherapeutic regimen(s) for advanced disease were treated with eribulin. Patients with eribulin-induced PN (new-onset PN or worsening of preexisting PN) were monitored until death or resolution of PN. Primary endpoints included the incidence, severity, and time to resolution of eribulin-induced PN. Secondary endpoints included time to disease progression and safety.

**Results:**

In this interim analysis (data cutoff date: July 1, 2019), 67 (32.4%) patients experienced any grade eribulin-induced PN, and 12 (5.8%) patients experienced grade ≥3 eribulin-induced PN. Median time to resolution of eribulin-induced PN was not reached. Median time to disease progression was 4.6 months (95% CI, 4.0-6.5). Treatment-emergent adverse events (TEAEs) occurred in 195 (93.8%) patients and serious TEAEs occurred in 107 (51.4%) patients.

**Conclusion:**

The rates of any grade and grade ≥3 eribulin-induced PN observed in this real-world study were consistent with those observed in phase III randomized clinical trials. No new safety findings were observed.

**Implications for Practice**

Peripheral neuropathy (PN), which involves damage to the peripheral nerves, is a common side effect of chemotherapy for breast cancer. The IRENE study reported on the incidence and resolution of PN observed in patients with locally advanced or metastatic breast cancer receiving eribulin, a chemotherapy agent, according to routine clinical care. The incidence of PN resulting from eribulin treatment was similar to that observed in clinical trials, while time to resolution of this side effect could not be measured. These results provide further information on PN resulting from eribulin treatment and may inform breast cancer treatment decisions.

## Introduction

Preferred treatment options for patients with advanced breast cancer who have been previously exposed to anthracyclines and taxanes include single-agent capecitabine, vinorelbine, or eribulin;^1^ however, these chemotherapy agents can lead to various cumulative toxicities, including peripheral neuropathy (PN).^2-4^ Although chemotherapy-induced PN is a well-known dose-limiting side effect, the nature of its persistence and resolution is less clear. Microtubule-targeting agents are among the most common classes of chemotherapeutic drugs used for the treatment of breast cancer; however, because microtubule function is critical to neuronal function, these agents are commonly associated with chemotherapy-induced PN.^5,6^

Eribulin, a synthetic analog of halichondrin B, is a halichondrin-class microtubule dynamics inhibitor with a binding site that is distinct from those of other microtubule-targeting agents.^2,7,8^ Eribulin is approved in the European Union for the treatment of patients with locally advanced or metastatic breast cancer whose disease progresses after at least one chemotherapeutic regimen for advanced disease (prior treatment should have included an anthracycline and a taxane in either the adjuvant or metastatic setting unless patients were not suitable for those treatments).^9^ Eribulin is also approved for the treatment of metastatic breast cancer after ≥2 prior lines of chemotherapy in the US^10^ and for the treatment of inoperable or recurrent breast cancer in Japan.^11^ EMBRACE, the pivotal randomized phase III clinical trial of eribulin versus treatment of physician’s choice in patients with previously treated locally recurrent or metastatic breast cancer, demonstrated significantly prolonged median overall survival in patients treated with eribulin compared with patients who received treatment of physician’s choice (13.1 months vs. 10.6 months, respectively; hazard ratio [HR], 0.81; 95% CI, 0.66-0.99; *P* = .041). Patients in the EMBRACE study treated with eribulin had a higher occurrence of PN (35%) than patients who received treatment of physician’s choice (16%). In addition, PN was the most common treatment-emergent adverse event (TEAE) leading to termination of eribulin, occurring in 5% of patients.^12^ Another randomized phase III trial comparing eribulin and capecitabine monotherapies demonstrated a similar rate of PN to that seen in the EMBRACE study (27.4% in patients treated with eribulin and 13.7% in patients treated with capecitabine).^13^

Although the studies described earlier provided valuable information about the incidence of eribulin-induced PN, data regarding the resolution of eribulin-induced PN are lacking. This noninterventional postauthorization safety study was designed to provide information on the frequency, severity, and time to resolution of eribulin-induced PN.

## Patients and Methods

### Study Design and Patients

IRENE is an ongoing observational, single-arm, prospective, multicenter, cohort study with a planned enrollment of about 400 patients across approximately 60 study sites in Germany. Adult patients with locally advanced or metastatic breast cancer whose disease progressed after 1-2 prior chemotherapeutic regimen(s) for advanced disease were treated with eribulin according to the Fachinformation (the German equivalent of Summary of Product Characteristics). Patients were ≥18 years of age at the time of informed consent and had received prior therapy with an anthracycline and a taxane in either the adjuvant or metastatic setting (unless they were not suitable for these treatments). Patients included in the study had received a maximum of 2 prior chemotherapeutic regimens for advanced disease (per protocol version no. 2.0, this criterion was changed to 1-3 prior chemotherapeutic regimens after enrollment of the patients included in this interim analysis), and the decision for patients to start treatment with eribulin was made prior to inclusion in the study.

Due to the observational nature of the study, the precise time point for data documentation was not specified but rather occurred during routine visits according to routine clinical care based on medical necessity. For PN-related endpoints, eribulin-induced PN included new-onset PN or worsening of preexisting PN during the study, as assessed by clinical examination and occurring any time after the first dose of eribulin until 30 days after the last dose of eribulin. Any new-onset PN or worsening of preexisting PN in patients who had not developed eribulin-induced PN at the time of the last eribulin dose and who initiated a new anticancer therapy with known neurotoxic potential within 30 days of the last eribulin dose were not considered as eribulin-induced PN events. Data related to PN were collected during the treatment period at the beginning of each eribulin treatment cycle. The planned observation period for each patient was approximately 15 months, and patients were monitored until death or resolution of PN (defined as complete resolution of PN or return to baseline levels). For all PN-related endpoints, PN included the Medical Dictionary for Regulatory Activities (version 22) preferred terms paresthesia, neuropathy peripheral, polyneuropathy, hypoesthesia, peripheral sensory neuropathy, muscular weakness, muscle spasms, and neuralgia.

Prior to study start, the observational study protocol along with the patient questionnaires, the patient information and informed consent form was presented to a competent ethics committee for assessment. Each participating physician was also able to seek advice from her/his competent ethics committee in line with professional legal obligations. All patients provided signed written informed consent prior to participation in this study.

### Endpoints

The primary endpoints included the number and proportion of patients with any eribulin-induced PN, severity of eribulin-induced PN (as determined by the Common Terminology Criteria for Adverse Events version 4.0 [CTCAE v4.0]), frequency of dose modifications or terminations of eribulin treatment due to eribulin-induced PN, time to eribulin termination due to eribulin-induced PN, frequency of resolution of eribulin-induced PN (defined as ended or returned to baseline, as determined by CTCAE v4.0 grade), time to resolution of eribulin-induced PN, and therapeutic interventions (eg, analgesics) used to treat eribulin-induced PN. Secondary endpoints included time to disease progression (defined as the time from the start of eribulin treatment to investigator assessment of clinical or radiological disease progression), safety (the number and proportion of patients experiencing non-serious and serious adverse drug reactions), and health-related quality of life scores (available at the time of the final analysis only).

### Statistical Methods

Due to the observational nature of the study, epidemiologic methods were employed for data analyses. Descriptive analyses of all collected data were performed. No formal statistical hypotheses were formulated, and no statistical tests were performed.

## Results

### Patients

At the time of this interim analysis (data cutoff date: July 1, 2019), 207 patients received ≥1 dose of eribulin and were included in the study. Among 69 patients who remained in the study at the data cutoff date, 29 patients were receiving eribulin treatment, 14 patients were undergoing baseline assessments, 17 patients had completed the end of treatment visit, 2 patients were in the off-treatment phase, and 7 patients were in the follow-up phase. Among the 165 patients who terminated eribulin treatment, the most common reason for termination was disease progression (*n* = 107), followed by adverse events (AEs; *n* = 33) and initiation of new anticancer treatment (*n* = 11). For 14 patients, “other” reasons for treatment termination were recorded (Supplementary Table S1).

Baseline demographic and clinical characteristics are shown in Table 1. Of 207 total patients, 183 (88.4%) had previously received neurotoxic anticancer treatment, 66 (31.9%) had a predisposition to PN (most commonly due to hypothyreosis [14.0%]), and 85 (41.1%) had PN ongoing at baseline.

**Table 1.** Baseline patient demographics and clinical characteristics.^a^

Characteristic	Patients^b^ (*N* = 207)
Age, years	
Median (range)	60.0 (32.0-83.0)
Mean (SD)	59.8 (11.4)
Sex, *n* (%)	
Female	206 (99.5)
Male	1 (0.5)
Mean body weight,^c^ kg (SD)	71.05 (16.36)
Locally advanced breast cancer, *n* (%)	14 (6.8)
Metastatic sites, *n* (%)	202 (97.6)
Bone	137 (66.2)
Brain	20 (9.7)
Lung	86 (41.5)
Liver	110 (53.1)
Other	95 (45.9)
Missing	16 (7.7)
Number of previous anticancer treatments, *n* (%)	202 (97.6)^d^
1	10 (4.8)
≥2	192 (92.8)
Type of previous anticancer treatment, *n* (%)^e^	202 (97.6)
Paclitaxel	134 (64.7)
Cyclophosphamide	100 (48.3)
Epirubicin	83 (40.1)
Bevacizumab	73 (35.3)
Capecitabine	66 (31.9)
Fulvestrant	61 (29.5)
Docetaxel	57 (27.5)
Tamoxifen	48 (23.2)
Letrozole	45 (21.7)
Previous neurotoxic anticancer treatment, *n* (%)	183 (88.4)
Taxanes	179 (86.5)
Platin derivatives	41 (19.8)
Vinca alkaloids	16 (7.7)
Other	12 (5.8)
Predisposition for peripheral neuropathy, *n* (%)	66 (31.9)
Hypothyreosis	29 (14.0)
Diabetes mellitus type 1 or 2	20 (9.7)
Renal impairment	10 (4.8)
Inflammatory diseases	5 (2.4)
Herpes zoster	4 (1.9)
Other	8 (3.9)
Peripheral neuropathy ongoing at baseline, *n* (%) Maximum CTCAE grade, *n*	85 (41.1)
1	56
2	24
3	5

^a^Data for all patients included in the PN and disease progression analyses are shown.

^b^Percentages in this column are based on the total number of patients (*N* = 207).

^c^ *n* = 202; 5 patients had missing data for this parameter.

^d^At the data cutoff date, previous anticancer treatment information was not available for 5 patients; thus, the percentage does not total to 100%.

^e^Previous anticancer therapies received by ≥20% of patients are shown.

Abbreviations: CTCAE: Common Terminology Criteria for Adverse Events; PN: peripheral neuropathy; SD: standard deviation.

#### Eribulin-Induced PN

**Figure 1.**
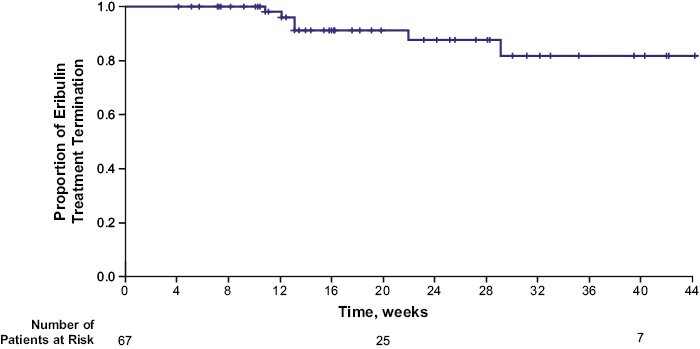
Kaplan-Meier plot for time to eribulin treatment termination due to eribulin-induced PN. Abbreviation: PN: peripheral neuropathy.

Frequencies of eribulin-induced PN events overall and those leading to dose modifications, delays, or treatment termination are listed in Table 2. Among the 207 patients included in the PN analyses, 67 (32.4%) experienced any grade eribulin-induced PN, and 32 (15.5%) experienced eribulin-induced worsening of preexisting PN. Twelve (5.8%) patients experienced grade ≥3 eribulin-induced PN (all events were grade 3). Seven of these 12 patients had improvement in the outcome of eribulin-induced PN over the course of the study; 3 had recovered, 3 were recovering, and 1 had recovered with sequelae. The median time to onset of eribulin-induced PN was 37.1 weeks (95% CI, 19.1-not reached); when examined in younger (<65 years) versus older (≥65 years) patients, median time to onset was 27.7 weeks and not reached, respectively. Dose modifications or delays due to an eribulin-induced PN event were uncommon, occurring in one patient and 2 patients, respectively. Six (2.9%) patients terminated eribulin treatment because of eribulin-induced PN. Patients <65 years of age experienced new-onset eribulin-induced PN, grade ≥3 eribulin-induced PN, and eribulin-induced PN leading to eribulin termination more frequently than patients ≥65 years of age (new-onset PN, 27.8% vs. 20.3%; grade ≥3 PN, 6.8% vs. 4.1%; and PN leading to eribulin termination, 3.8% vs. 1.4%). Median time to eribulin treatment termination due to eribulin-induced PN was not reached (Fig. 1).

**Table 2.** Patients who experienced eribulin-induced PN.^a^

Incidence, *n* (%)	Total (*N* = 207)	Age <65 years (*n* = 133)	Age ≥65 years (*n* = 74)
Any eribulin-induced PN event^b^	67 (32.4)	45 (33.8)	22 (29.7)
Worsening of preexisting PN	32 (15.5)	21 (15.8)	11 (14.9)
New-onset PN	52 (25.1)	37 (27.8)	15 (20.3)
Any eribulin-induced PN event of grade ≥3	12 (5.8)	9 (6.8)	3 (4.1)
Resolution of all eribulin-induced PN events^c^^,^^d^^,^^e^	9 (13.4)	8 (17.8)	1 (4.5)
Any therapeutic intervention for eribulin-induced PN^e^^,^^f^	12 (17.9)	9 (20.0)	3 (13.6)
Any eribulin-induced PN event leading to dose modification^g^	1 (0.5)	1 (0.8)	0
Any eribulin-induced PN event leading to dose delay^g^	2 (1.0)	2 (1.5)	0
Any eribulin-induced PN event leading to eribulin termination	6 (2.9)	5 (3.8)	1 (1.4)

^a^Data for all patients included in the PN and disease progression analyses are shown.

^b^Patients appear in each category in which they had ≥1 event.

^c^Resolution is defined as all ongoing PN events resolved or, in the case of worsening PN, returned to baseline levels.

^d^Median time to resolution of eribulin-induced PN was not reached.

^e^Percentages are based on the number of patients with any eribulin-induced PN event.

^f^The most common interventions were vitamin B12 (*n* = 4), pyridoxine, thiamin (*n* = 3,), ascorbic acid, biotin, cocarboxylase (*n* = 2,), and pyridoxine hydrochloride (*n* = 2).

^g^A dose modification is defined as a change in administered dose between 2 study visits. A dose delay is defined as a period of more than 7 days between day 1 and day 8 within a treatment cycle or as a period of more than 14 days between day 8 and day 1 of the following treatment cycle.

Abbreviations: PN: peripheral neuropathy.

**Figure 2.**
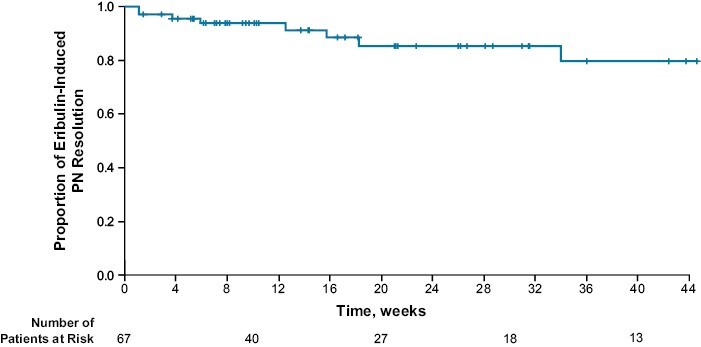
Kaplan-Meier plot for time to resolution of eribulin-induced PN. (**A**) If peripheral neuropathy was not resolved at the time of analysis, time to resolution of PN was censored at the earliest date of the following events: date of study termination or date of data cutoff (for interim analysis). (**B**) Of the 67 patients with eribulin-induced PN, 58 (86.6%) patients were censored at the data cutoff date. The most common reason for censoring was death (24 out of 67 patients; 35.8%). Abbreviation: PN: peripheral neuropathy.

Among the 67 patients who had an eribulin-induced PN event, 9 (13.4%) experienced event resolution (defined as all ongoing PN events resolved or, in the case of worsening PN, returned to baseline levels). Median time to resolution of eribulin-induced PN was not reached (Fig. 2). Among the 67 patients with eribulin-induced PN, 58 (86.6%) patients were censored at the data cutoff date. The most common reason for censoring was death (24 of 67 patients; 35.8%). Twelve patients received a therapeutic intervention for eribulin-induced PN (the most common interventions were vitamin B12 [*n* = 4]; pyridoxine, thiamin [*n* = 3]; ascorbic acid, biotin, cocarboxylase [*n* = 2]; and pyridoxine hydrochloride [*n* = 2]).

### Time to Disease Progression

**Figure 3.**
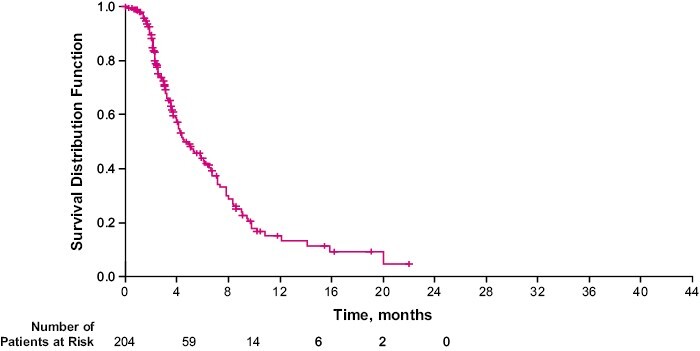
Kaplan-Meier estimate of time to disease progression. (**A**) Time to progression was defined as the time from the first dose of eribulin until tumor progression, determined either radiographically or by clinical examination. Deaths (without documented disease progression) were censored at the time of death. Three patients were excluded from this analysis due to contradictory values (date of disease progression before date of first dose of eribulin). (**B**) 116 patients (of 204 patients, 56.9%) experienced disease progression. Median time to disease progression was 4.6 months (95% CI, 4.0-6.5). Abbreviation: CI: confidence interval.

During the study and until the data cutoff date, 116 patients (of 204 patients; 56.9%) experienced disease progression. Median time to disease progression was 4.6 months (95% CI, 4.0-6.5 [Fig. 3]).

### Safety

An erratum report from June 16, 2021 that encompassed only the TEAE analyses corrected the total number of patients who received ≥1 dose of eribulin to 208. Thus, all TEAE percentages were based on a total of 208 patients. A summary of TEAEs is presented in Table 3. TEAEs of any grade and grade ≥3 occurred in 195 (93.8%) and 129 (62.0%) patients, respectively. Eribulin-related TEAEs were reported in 148 (71.2%) patients. Nonfatal and fatal serious TEAEs occurred in 86 (41.3%) and 41 (19.7%) patients, respectively; eribulin-related serious TEAEs occurred in 33 (15.9%) patients. Eribulin-related TEAEs led to eribulin dose modifications in 23 (11.1%) patients and to eribulin termination in 13 (6.3%) patients. When comparing TEAE rates between younger (<65 years) and older (≥65 years) patients, similar rates of overall TEAEs and eribulin-related TEAEs were reported; additionally, similar rates of related TEAEs leading to eribulin dose modification and treatment termination were reported in younger and older patients. As shown in Table 4, the most common TEAEs reported as being at least possibly related to eribulin included leukopenia (15.4%) and alopecia, fatigue, and neutropenia (14.9% each).

**Table 3.** Summary of TEAEs.^a^

AEs, *n* (%)	Total (*N* = 208)	Age <65 years (*n* = 134)	Age ≥65 years (*n* = 74)
Any TEAE	195 (93.8)	126 (94.0)	69 (93.2)
Eribulin-related TEAE	148 (71.2)	95 (70.9)	53 (71.6)
TEAE grade ≥3	129 (62.0)	85 (63.4)	44 (59.5)
Serious TEAE	107 (51.4)	70 (52.2)	37 (50.0)
Fatal^b^	41 (19.7)	28 (20.9)	13 (17.6)
Nonfatal	86 (41.4)^c^	56 (41.8)	30 (40.5)
Serious eribulin-related TEAE	33 (15.9)	20 (14.9)	13 (17.6)
Eribulin-related TEAEs leading to dose modification	23 (11.1)	16 (11.9)	7 (9.5)
Eribulin-related TEAEs leading to eribulin termination	13 (6.3)	7 (5.2)	6 (8.1)

^a^TEAEs are based on a total of 208 patients (per an erratum report that corrected the total number of patients who received ≥1 dose of eribulin to 208).

^b^Four patients died due to five grade 5 events judged by the investigator to be at least possibly related to eribulin. The respective events were infection, malignant neoplasm progression, pneumonia, fatigue, and stomatitis.

^c^Due to a coding error in the Safety Erratum Report dated June 16, 2021, the total percentage of nonfatal serious TEAEs is listed as 41.4% rather than 41.3%.

Abbreviations: AE: adverse event; TEAE: treatment-emergent adverse event.

**Table 4.** Most common eribulin-related TEAEs (>5% any grade).^a^^,^^b^^,^^c^

Adverse events, *n* (%)	Total (*N* = 208)		
	Any grade	Grade 3	Grade 4
Leukopenia	32 (15.4)	19 (9.2)^d^	2 (1.0)
Alopecia	31 (14.9)	4 (1.9)	0
Fatigue	31 (14.9)	7 (3.4)	0
Neutropenia	31 (14.9)	10 (4.8)	4 (1.9)
Nausea	18 (8.7)	2 (1.0)	0
Paresthesia	16 (7.7)	4 (1.9)	0
Polyneuropathy	16 (7.7)	3 (1.4)	0
Hypoesthesia	14 (6.8)^d^	4 (1.9)	0
Peripheral neuropathy	13 (6.3)	2 (1.0)	0
Peripheral sensory neuropathy	13 (6.3)	2 (1.0)	0

^a^TEAEs are based on a total of 208 patients (per an erratum report that corrected the total number of patients who received ≥1 dose of eribulin to 208).

^b^TEAEs reported as at least possibly related to eribulin are included. Severity was graded according to CTCAE version 4.0. At each level of patient summarization, only the most severe event was counted if the patient reported more than one event. If CTCAE grade of TEAE was missing, the adverse event is only included in the column labeled “Any grade.”

^c^Coded with Medical Dictionary for Regulatory Activities version 22.0.

^d^Due to a coding error, these values were derived from a population of 207 patients rather than the corrected number of patients in the safety population per the Safety Erratum Report dated June 16, 2021 (*n* = 208), resulting in slightly higher percentages.

Abbreviations: CTCAE: Common Terminology Criteria for Adverse Events; TEAE: treatment-emergent adverse event.

## Discussion

This observational study assessed the incidence and resolution of eribulin-induced PN in patients with locally advanced or metastatic breast cancer and demonstrated consistency between the frequency of eribulin-induced PN events in real-world and randomized phase III clinical trial settings. The IRENE interim analysis demonstrated a 32.4% overall eribulin-induced PN rate and a 5.8% grade ≥3 eribulin-induced PN rate; results were similar to the EMBRACE trial of eribulin versus treatment of physician’s choice in patients with previously treated locally recurrent or metastatic breast cancer, which demonstrated a 35% overall PN rate, an 8% grade 3 PN rate, and a <1% grade 4 PN rate.^12^ Similarly, a randomized phase III clinical trial comparing eribulin with capecitabine in patients with previously treated locally advanced or metastatic breast cancer (Study 301) demonstrated an overall PN incidence of 27.4%, a grade 3 PN incidence of 6.4%, and a grade 4 PN incidence of 0.6%.^13^ Similarities between these randomized trials and our study were observed despite the higher median age of patients in the IRENE study (60.0 years [range, 32.0-83.0 years]) as compared with both the EMBRACE study (55.0 years [range, 28-85 years])^12^ and Study 301 (54.0 years [range, 24-80 years]).^13^ All study comparisons should be made with caution, as variations in PN classification may have occurred (eg, the aforementioned randomized phase III clinical trials used CTCAE v3.0 to classify PN, while we used CTCAE v4.0).

Furthermore, our results are consistent with a post-marketing observational study of eribulin-induced PN in Japanese patients without PN at baseline (mean age, 58.4 years [range, 32-83 years]); in this study, 28.1% of patients experienced PN during eribulin treatment (1.1% of patients experienced grade 3 PN).^14^ Although a higher percentage of patients in the IRENE study experienced grade ≥3 eribulin-induced PN, more than half of these patients also experienced improvements in severity of PN events over the course of the study. Additionally, a higher percentage of patients in the IRENE study had received ≥2 prior lines of therapy compared with those in the Japanese observational study (92.8% vs. 40.6%). Thus, the incidence of eribulin-induced PN in our study was consistent with results shown in both controlled and observational treatment settings.

At the time of data cutoff, we were unable to quantify the time to resolution of PN; however, the incidence and resolution of eribulin-induced PN continue to be assessed in this ongoing study. Notably, only 17.9% of the 67 patients with eribulin-induced PN in our study received a therapeutic intervention for PN. The low number of patients who were treated for eribulin-induced PN may be attributed to the limited number of effective treatments for chemotherapy-induced PN that do not interfere with the antitumor effects of chemotherapy.^15^ Additionally, although certain eribulin-induced PN events (ie, new onset of eribulin-induced PN, eribulin-induced PN grade ≥3, and eribulin-induced PN leading to eribulin termination) occurred at a slightly higher frequency in younger (<65 years) versus older (≥65 years) patients, these differences should be interpreted with caution due to the observational and descriptive nature of this study.

In addition to consistency between PN incidence in previous studies and the IRENE study, there were no new safety findings observed in this study, and the reported AEs were consistent with the known drug safety profile of eribulin.^9,12,13^ The overall rate of TEAEs in our study (93.8%) is consistent with rates reported in EMBRACE (99%) and Study 301 (94.1%). The rate of serious TEAEs for patients receiving eribulin in our study (51.4%) is notably higher than rates observed in EMBRACE (25%) and Study 301 (17.5%); the higher median age of patients in our study should be considered when comparing these results. Although the observational and descriptive nature of IRENE somewhat limits its generalizability to previous studies, the 55 study sites that enrolled patients until the data cutoff date were spread across Germany, with all but 3 study sites enrolling <10 patients each. Although this widespread enrollment strategy served to eliminate site-specific bias as much as possible, the noninterventional, observational nature of this study must be considered when interpreting the results.

## Conclusion

In this interim analysis of the real-world data derived from the IRENE study, the incidence of eribulin-induced PN was consistent with incidences reported in randomized phase III clinical trials of patients with metastatic breast cancer. In addition, the safety findings of the current analysis were consistent with the known safety profile of eribulin. Even though we could not draw any conclusions about the time to resolution of PN from this interim analysis, we demonstrated that the median time to resolution of PN was not reached. Thus, this study is ongoing, with final results expected in April 2023.

## Supplementary Material

oyad191_suppl_Supplementary_Table_S1

## Data Availability

The data underlying this article are available in the article and in Supplementary Material.
