# Supplementary material for: Incidence and Resolution of Eribulin-Induced Peripheral Neuropathy (IRENE) in Locally Advanced or Metastatic Breast Cancer: Prospective Cohort Study
Source: Oncologist. 2023 Aug 9;28(12):e1152–9. doi: 10.1093/oncolo/oyad191 (PMC10712709; doi:10.1093/oncolo/oyad191)
Supplement: oyad191_suppl_Supplementary_Table_S1 [file oyad191_suppl_supplementary_table_s1.docx]

**Supplemental Table 1**. Patient Disposition

| **Category** | ***n*** |
| --- | --- |
| Screened^a^ | 232 |
| Treated with eribulin^b^ | 207 |
| Ongoing in study^c^ | 69 |
| Receiving eribulin treatment | 29 |
| Undergoing baseline assessments | 14 |
| Completed end-of-treatment visit | 17 |
| In off-treatment phase | 2 |
| In follow-up phase | 7 |
| Terminated eribulin treatment^d^ | 165 |
| Disease progression | 107 |
| Adverse events | 33 |
| Initiation of new anticancer treatment | 11 |
| Other | 14 |

^a^Of 232 screened patients, 1 was a screen failure.
^b^Includes patients who received ≥1 dose of eribulin at the time of data cutoff (1 July 2019).
^c^Includes patients who were in the study at the time of data cutoff.
^d^Includes patients who terminated treatment by the data cutoff date.
